# Geographic Differences in Sex-Specific Chronic Obstructive Pulmonary Disease Mortality Rate Trends Among Adults Aged ≥25 Years — United States, 1999–2019

**DOI:** 10.15585/mmwr.mm7118a1

**Published:** 2022-05-06

**Authors:** Susan A. Carlson, Anne G. Wheaton, Kathleen B. Watson, Yong Liu, Janet B. Croft, Kurt J. Greenlund

**Affiliations:** 1 Division of Population Health, National Center for Chronic Disease Prevention and Health Promotion, CDC.

Chronic obstructive pulmonary disease (COPD) accounts for the majority of deaths from chronic lower respiratory diseases, the fourth leading cause of death in the United States in 2019.* COPD mortality rates are decreasing overall. Although rates in men remain higher than those in women, declines have occurred among men but not women (*1*). To examine the geographic variation in sex-specific trends in age-adjusted COPD mortality rates among adults aged ≥25 years, CDC analyzed 1999–2019 death certificate data, by urban-rural status,^†^ U.S. Census Bureau region,^§^ and state. Among women, no significant change in overall COPD mortality occurred during this period; however, rates increased significantly in small metropolitan (average annual percent change [AAPC] = 0.6%), micropolitan (1.2%), and noncore (1.9%) areas and in the Midwest (0.6%). Rates decreased significantly in large central (−0.9%) and fringe metropolitan (−0.4%) areas (and in the Northeast (−0.5%) and West (−1.2%). Among men, rates decreased significantly overall (−1.3%), in all urban-rural areas (range = −1.9% [large central metropolitan] to −0.4% [noncore]) and in all regions (range = −2.0% [West] to −0.9% [Midwest]). Strategies to improve the prevention, treatment, and management of COPD are needed, especially to address geographic differences and improve the trend in women, to reduce COPD deaths.

Mortality data from the CDC National Vital Statistics System during 1999–2019 were analyzed to determine the number and rate of deaths from COPD among adults aged ≥25 years for each year by sex and by geographic characteristics of the person's place of legal residence at the time of death.^¶^ Each death certificate identifies a single underlying cause; COPD was identified using *International Classification of Diseases, Tenth Revision* codes J40–J44. Queries to CDC WONDER generated yearly age-adjusted sex-specific mortality rates (deaths per 100,000 standard population) by urban-rural status, U.S. Census Bureau region, and state. Age-adjusted rates with 95% CIs were estimated using the 2000 U.S. standard population and 10-year age groups. Trends were evaluated using Joinpoint software (version 4.8.0.1; National Cancer Institute).** Annual percent change (APC) for each trend segment and AAPC from 1999 to 2019 were estimated; values significantly <0 (p≤0.05) were interpreted as a significant decrease in mortality rates, and values significantly >0 were interpreted as a significant increase. This activity was reviewed by CDC and was conducted consistent with applicable federal law and CDC policy.^††^

Differences between men and women in annual age-adjusted COPD mortality rates were smaller in 2019 (62.8 versus 53.0, respectively) than in 1999 (88.2 versus 54.6, respectively)(Table). COPD mortality rates among men in 1999 and 2019 and among women in 2019 were inversely related to urbanicity; no clear urban-rural pattern was observed among women in 1999. Similar to region-specific patterns observed among men in 1999 and 2019, age-adjusted COPD mortality rates among women in 2019 were lowest in the Northeast, followed by the West, and highest in the South and Midwest; in 1999, rates among women were highest in the West.

**TABLE:** Sex-specific chronic obstructive pulmonary disease related deaths and age-adjusted mortality rates* among adults aged ≥25 years and trends in mortality rates, by geographic characteristics — United States, 1999–2019

Geographic characteristic	1999		2019		AAPC^†^ 1999–2019 (95% CI)	No. of joinpoints	Segment-specific APC^† ^1999–2019 (95% CI)
	No. of deaths	Deaths per 100,000 population (95% CI)	No. of deaths	Deaths per 100,000 population (95% CI)			
**Women**							
**Overall**	**58,040**	**54.6 (54.1 to 55.0)**	**80,422**	**53.0 (52.6 to 53.4)**	**0.1 (−0.1 to 0.3)**	**0**	**—^§^**
**Urban-rural status^¶^**							
Large central metropolitan	15,833	52.3 (51.4 to 53.1)	16,919	40.1 (39.5 to 40.7)	−0.9 (−1.2 to −0.7)**	0	—
Large fringe metropolitan	13,006	54.9 (54.0 to 55.9)	18,337	48.8 (48.1 to 49.5)	−0.4 (−0.6 to −0.2)**	0	—
Medium metropolitan	12,334	56.0 (55.0 to 57.0)	18,010	55.3 (54.5 to 56.1)	−0.2 (−0.9 to 0.5)	1	1999–2017: 0.3 (0.0 to 0.5)** 2017–2019: −4.2 (−10.7 to 2.7)
Small metropolitan	6,067	58.7 (57.2 to 60.2)	9,485	64.5 (63.2 to 65.9)	0.6 (0.4 to 0.8)**	0	—
Micropolitan (nonmetropolitan)	6,206	56.8 (55.4 to 58.2)	9,924	71.3 (69.8 to 72.7)	1.2 (0.7 to 1.7)**	1	1999–2015: 1.6 (1.3 to 1.9)** 2015–2019: −0.5 (−2.7 to 1.8)
Noncore (nonmetropolitan)	4,594	51.5 (50.0 to 53.3)	7,747	73.8 (72.1 to 75.4)	1.9 (1.5 to 2.4)**	1	1999–2011: 2.5 (2.0 to 3.1)**^ ^2011–2019: 1.0 (0.1 to 2.0)**
**U.S. Census Bureau region^††^**							
Northeast	11,163	48.1 (47.2 to 49.0)	12,250	42.1 (41.3 to 42.8)	−0.5 (−0.7 to −0.3)**	0	—
Midwest	14,028	54.9 (54.0 to 55.8)	19,234	58.9 (58.0 to 59.7)	0.6 (0.0 to 1.1)**	1	1999–2013: 1.1 (0.7 to 1.6)** 2013–2019: −0.7 (−2.2 to 0.8)
South	20,319	54.5 (53.7 to 55.2)	33,644	59.3 (58.6 to 59.9)	0.3 (−0.3 to 1.0)	1	1999–2017: 0.8 (0.5 to 1.0)** 2017–2019: −3.4 (−9.4 to 3.1)
West	12,530	61.6 (60.5 to 62.7)	15,294	46.0 (45.3 to 46.7)	−1.2 (−1.4 to −1.0) **	0	—
**Men**							
**Overall**	**60,416**	**88.2 (87.4 to 88.9)**	**71,991**	**62.8 (62.3 to 63.2)**	−1.3 (−1.5 to −1.1)**	**0**	**—**
**Urban-rural status^¶^**							
Large central metropolitan	14,618	77.7 (76.5 to 79.0)	14,452	48.0 (47.2 to 48.8)	−1.9 (−2.1 to −1.7)**	0	—
Large fringe metropolitan	11,981	79.1 (77.6 to 80.5)	15,122	54.2 (53.3 to 55.1)	−1.6 (−1.8 to −1.4)**	0	—
Medium metropolitan	13,092	91.0 (89.5 to 92.6)	16,194	64.8 (63.8 to 65.8)	−1.3 (−1.5 to −1.1)**	0	—
Small metropolitan	6,786	99.8 (97.4 to 102.2)	8,706	75.5 (73.9 to 77.2)	−1.0 (−1.2 to −0.8)**	0	—
Micropolitan (nonmetropolitan)	7,433	102.5 (100.2 to 104.9)	9,641	87.0 (85.2 to 88.8)	−0.6 (−0.8 to −0.5)**	0	—
Noncore (nonmetropolitan)	6,506	106.6 (104.0 to 109.2)	7,876	90.2 (88.2 to 92.2)	−0.4 (−0.6 to −0.1)**	0	—
**U.S. Census Bureau region^††^**							
Northeast	10,574	75.6 (74.1 to 77.0)	10,187	49.5 (48.6 to 50.5)	−1.8 (−2.0 to −1.5)**	0	—
Midwest	14,886	92.3 (90.8 to 93.8)	17,398	70.7 (69.6 to 71.7)	−0.9 (−1.2 to −0.7)**	0	—
South	22,415	92.4 (91.2 to 93.7)	29,956	69.0 (68.2 to 69.8)	−1.1 (−1.3 to −0.9)**	0	—
West	12,541	88.6 (87.0 to 90.2)	14,450	55.6 (54.7 to 56.5)	−2.0 (−2.2 to −1.8)**	0	—

**Abbreviations**: AAPC = average annual percent change; APC = annual percent change; COPD = chronic obstructive pulmonary disease.

* Per 100,000 standard population. Age-adjusted COPD mortality rates were calculated using the 2000 U.S. Census Bureau projected population and 10-year age groups.

^†^ COPD trends were assessed as the AAPC from 1999 to 2019 and as the APC for segment-specific periods when a joinpoint was detected.

^§^ Dashes indicate that the best-fit joinpoint model did not include any trend segments.

^¶^ As defined in the CDC National Center for Health Statistics 2013 Urban-Rural Classification Scheme for Counties with six urbanization levels: four metropolitan (large central metropolitan, large fringe metropolitan, medium metropolitan, and small metropolitan) and two nonmetropolitan (micropolitan and noncore). https://www.cdc.gov/nchs/data/series/sr_02/sr02_166.pdf

** Significantly different from 0 at p≤0.05. For APCs and for AAPCs within one segment (e.g., no joinpoint), the t-distribution is used. For AAPCs within multiple segments (e.g., one joinpoint), the normal (z) distribution is used.

^††^ U.S. Census Bureau regions: *Northeast*: Connecticut, Maine, Massachusetts, New Hampshire, New Jersey, New York, Pennsylvania, Rhode Island, and Vermont. *Midwest*: Illinois, Indiana, Iowa, Kansas, Michigan, Minnesota, Missouri, Nebraska, North Dakota, Ohio, South Dakota, and Wisconsin. *South*: Alabama, Arkansas, Delaware, District of Columbia, Florida, Georgia, Kentucky, Louisiana, Maryland, Mississippi, North Carolina, Oklahoma, South Carolina, Tennessee, Texas, Virginia, and West Virginia. *West*: Alaska, Arizona, California, Colorado, Hawaii, Idaho, Montana, Nevada, New Mexico, Oregon, Utah, Washington, and Wyoming. https://www2.census.gov/geo/pdfs/maps-data/maps/reference/us_regdiv.pdf

During 1999–2019, overall age-adjusted COPD mortality rates among women did not change significantly, whereas rates among men decreased significantly (AAPC = −1.3%). However, trends in age-adjusted, sex-specific COPD mortality rates differed by region and urban-rural status during this period (Figure 1). Among women, rates increased significantly in the Midwest (0.6%), did not change significantly in the South, and decreased significantly in the Northeast (−0.5%) and West (−1.2%). The change in rates among women was not significant when analysis was limited to later years in the Midwest (2013–2019), but was significant in the South (0.8%) when analysis was limited to 1999–2017. Among men, significant decreases were observed from 1999 to 2019 in all regions (range = −2.0% [West] to −0.9% [Midwest]). By urban-rural status, among women, the COPD mortality rate decreased significantly from 1999 to 2019 in large central (−0.9%) and fringe (−0.4%) metropolitan areas, did not change significantly in medium metropolitan areas, and increased significantly in small metropolitan (0.6%), micropolitan (1.2%), and noncore (1.9%) areas. The change in COPD mortality rates among women was not significant when analysis was limited to later years in medium metropolitan (2017–2019) and in micropolitan areas (2015–2019); the increase in rates slowed in noncore areas after 2011. COPD mortality rates among men decreased in all urban-rural categories (range = −1.9% [large central metropolitan] to −0.4% [noncore]).

**FIGURE 1:**
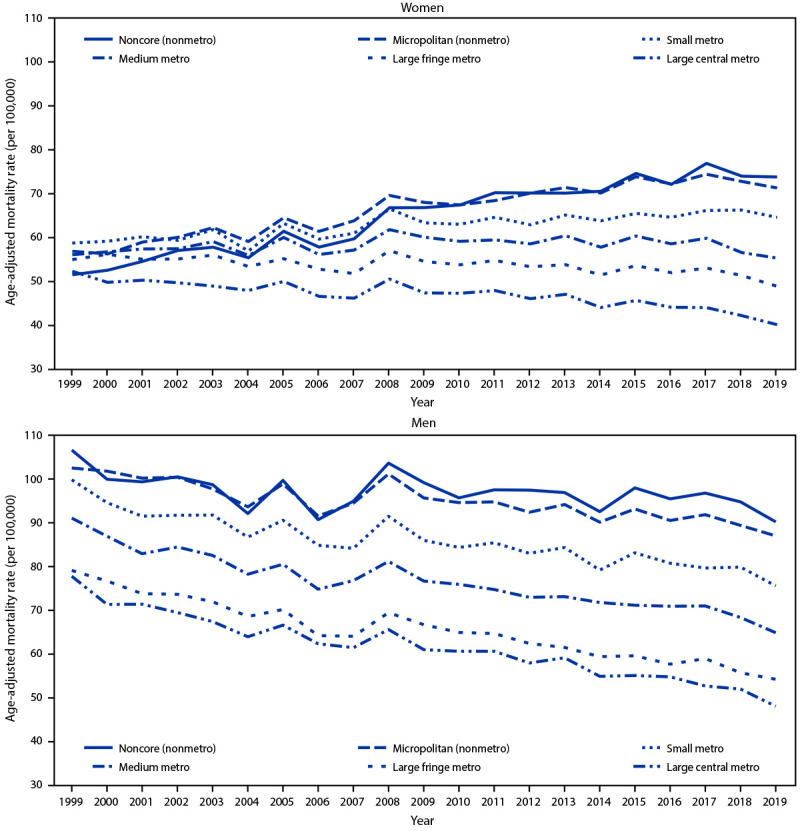
Sex-specific trends in age-adjusted chronic obstructive pulmonary disease mortality rates among adults aged ≥25 years,* by urban-rural status^†^ — United States, 1999–2019 **Abbreviation**: COPD = chronic obstructive pulmonary disease. * Per 100,000 population. Age-adjusted COPD mortality rates were calculated using the 2000 U.S. Census Bureau projected population and 10-year age groups. ^†^ As defined in the CDC National Center for Health Statistics 2013 Urban-Rural Classification Scheme for Counties with six urbanization levels: four metropolitan (large central metro, large fringe metro, medium metro, and small metro) and two nonmetropolitan (micropolitan and noncore). https://www.cdc.gov/nchs/data/series/sr_02/sr02_166.pdf

Among women, rates decreased significantly in 17 states (AAPC range = −1.9% [California] to −0.4% [New Jersey and Arizona]) and increased significantly in 18 states (range = 0.4% [Wisconsin] to 2.9% [Arkansas]) (Figure 2) (Supplementary Table; https://stacks.cdc.gov/view/cdc/116406). Among men, rates decreased significantly in 45 states and the District of Columbia (range = −4.2% [Alaska] to −0.3% [Indiana]) and increased significantly in Arkansas (0.5%). State-level mortality rates among women ranged from 24.0 (Hawaii) to 93.9 (Wyoming) in 1999 and 16.7 (Hawaii) to 89.8 (West Virginia) in 2019. Among men, rates ranged from 41.9 (Hawaii) to 143.2 (Wyoming) in 1999 and from 30.4 (District of Columbia) to 104.0 (Oklahoma) in 2019.

**FIGURE 2:**
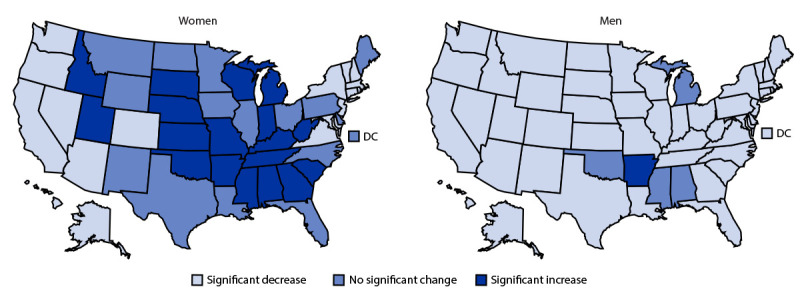
State-level changes* in sex-specific age-adjusted chronic obstructive pulmonary disease mortality rates† among adults aged ≥25 years — United States, 1999–2019 **Abbreviations:** AAPC = average annual percent change; COPD = chronic obstructive pulmonary disease; DC = District of Columbia. * Statistically significant changes were determined using the estimated AAPC with all years included (1999–2019). AAPCs significantly <0 were interpreted as a significant decrease while those significantly >0 were interpreted as a significant increase. ^^†^^ Per 100,000 population. Age-adjusted COPD mortality rates were calculated using the 2000 U.S. Census Bureau projected population and 10-year age groups.

## Discussion

Age-adjusted COPD mortality rates decreased among men from 1999 to 2019; however, rates remained higher in men than women. Among women, although overall rates exhibited no significant change, rates increased among some geographic subgroups, including women living in the Midwest and those living in small metropolitan or nonmetropolitan areas. Among both men and women, urban-rural disparities became more pronounced during this time. Efforts are needed to continue the decreasing trend in COPD mortality rates among men and improve the trend among women. Findings highlight several important geographical areas to focus COPD prevention (e.g., smoking cessation), early diagnosis, treatment (e.g., medication and oxygen therapy), and management strategies (e.g., pulmonary rehabilitation; efforts to slow declining lung function, improve exercise tolerance, and prevent exacerbations).

COPD mortality might differ by sex for several reasons. First, tobacco smoking is the main cause of COPD in the United States, and cigarette smoking declined first among men (since the 1960s) and later among women (since the 1980s) (*2*). Second, women might be more vulnerable to the effects of tobacco (*2*–*4*). Third, women account for most patients with COPD who have never smoked, suggesting that women might be more susceptible to secondhand smoke or nonsmoking-related factors (*3*,*5*,*6*). Fourth, disease presentation and rates of exacerbations might differ by sex which can result in delayed diagnosis and higher rates of exacerbations in women (*3*,*4*). Finally, women with COPD also face challenges related to their interactions with the health care system (*3*). Women face higher rates of misdiagnosis or delayed diagnosis that can potentially lead to suboptimal treatment (*3*,*4*). Improving understanding about the reasons for the increasing COPD mortality rates in certain subgroups of women can help guide the development and implementation of prevention, early diagnosis, treatment, and management strategies that are specifically tailored for women.

Region-specific patterns in COPD mortality in 2019 were similar among men and women (e.g., highest in the Midwest and South), and urban-rural disparities became more pronounced among both women and men during the past 20 years. For example, in 1999 there was no significant difference in rates between large central metropolitan areas and noncore areas among women, but in 2019 the rate was 84% higher in noncore areas. Similarly, for men the relative difference between these two areas increased from 37% in 1999 to 88% in 2019. These findings update previous studies that examined geographic differences in COPD prevalence and mortality (*7*,*8*). The COPD National Action Plan^§§^ provides a comprehensive framework for developing and implementing COPD prevention, treatment, and management strategies. Developing strategies that maximize the use of setting-specific resources (e.g., engaging existing stakeholders as well as providing patient-centric clinical guidelines to health care providers most likely to deliver COPD care within a setting) and help adults overcome setting-specific challenges are important in reducing urban-rural, regional, and state-level disparities in COPD mortality overall (*9*). For example, adults in rural areas might be more likely to experience challenges related to access (e.g., less access to pulmonologists and longer travel distances to health care facilities) (*10*) and cost (e.g., higher likelihood of being uninsured or having a lower socioeconomic status).^¶¶^

The findings in this report are subject to at least two limitations. First, COPD mortality might be underestimated because adults with COPD are more likely to have comorbidities (e.g., cardiovascular disease, stroke, diabetes, or cancer) (*1**,**8*) that might displace COPD as the underlying cause reported on the death certificate. Second, the 2013 CDC National Center for Health Statistics Urban-Rural Classification Scheme for Counties is well-suited to assessing and monitoring health differences across the full urbanization continuum; however, the assumption that the six urban-rural classifications reflect consistent types of distinct populations and social environments within and across each state could be an oversimplification.

Continued efforts are needed to prevent COPD and support early diagnosis, treatment (e.g., medication and oxygen therapy), and management (e.g., pulmonary rehabilitation). In addition, strategies that help improve the trend among women and address geographic differences have the potential to reduce COPD mortality.

**Summary**

**What is already known about this topic?**

Chronic obstructive pulmonary disease (COPD) accounts for most deaths from chronic lower respiratory diseases, the fourth leading cause of death in 2019 in the United States. COPD mortality rates are decreasing overall.

**What is added by this report?**

From 1999 to 2019, overall age-adjusted COPD mortality rates among women did not change; however, rates increased among women living in the Midwest and those in small metropolitan or nonmetropolitan areas. COPD mortality rates are higher among men; however, rates decreased overall and among all regional and urban-rural subgroups.

**What are the implications for public health practice?**

To reduce COPD deaths, strategies to improve the prevention, treatment, and management of COPD are needed, especially strategies that address geographic differences and improve the trend among women.

## Footnotes

*
https://www.cdc.gov/nchs/data/nvsr/nvsr70/nvsr70-08-508.pdf

† As defined in the CDC National Center for Health Statistics 2013 Urban-Rural Classification Scheme for Counties with six urbanization levels: four metropolitan (large central metropolitan, large fringe metropolitan, medium metropolitan, and small metropolitan) and two nonmetropolitan (micropolitan and noncore). https://www.cdc.gov/nchs/data/series/sr_02/sr02_166.pdf

§ U.S. Census Bureau regions: *Northeast*: Connecticut, Maine, Massachusetts, New Hampshire, New Jersey, New York, Pennsylvania, Rhode Island, and Vermont. *Midwest*: Illinois, Indiana, Iowa, Kansas, Michigan, Minnesota, Missouri, Nebraska, North Dakota, Ohio, South Dakota, and Wisconsin. *South*: Alabama, Arkansas, Delaware, District of Columbia, Florida, Georgia, Kentucky, Louisiana, Maryland, Mississippi, North Carolina, Oklahoma, South Carolina, Tennessee, Texas, Virginia, and West Virginia. *West*: Alaska, Arizona, California, Colorado, Hawaii, Idaho, Montana, Nevada, New Mexico, Oregon, Utah, Washington, and Wyoming. https://www2.census.gov/geo/pdfs/maps-data/maps/reference/us_regdiv.pdf

¶ The analysis focused on the adult population because COPD mortality is rare in the non-adult population. The analysis was restricted to adults aged ≥25 years because standard age-adjusted rates (calculated with standard populations) are only available from CDC WONDER using 10-year age groups (<1, 1–4, 5–14, 15–24, 25–34, 35–44, 45–54, 55–64, 65–74, 75–84, and ≥85 years). https://wonder.cdc.gov/

** Joinpoint software identifies statistically significant changes in a trend using Monte Carlo permutation, then fits them as a series of joined trend segments. https://surveillance.cancer.gov/joinpoint/

†† 45 C.F.R. part 46, 21 C.F.R. part 56; 42 U.S.C. Sect. 241(d); 5 U.S.C. Sect. 552a; 44 U.S.C. Sect. 3501 et seq.

§§
https://www.nhlbi.nih.gov/health-topics/all-publications-and-resources/copd-national-action-plan

¶¶
https://www.ruralhealthweb.org/about-nrha/about-rural-health-care
